# Identification of Bladder Cancer Subtypes Based on Necroptosis-Related Genes, Construction of a Prognostic Model

**DOI:** 10.3389/fsurg.2022.860857

**Published:** 2022-04-11

**Authors:** Shiwen Nie, Youlong Huili, Yadong He, Junchao Hu, Shaosan Kang, Fenghong Cao

**Affiliations:** ^1^Department of Urology, North China University of Science and Technology Affiliated Hospital, Tangshan, China; ^2^Department of General Practice, North China University of Science and Technology Affiliated Hospital, Tangshan, China

**Keywords:** necroptosis, bladder cancer, prognostic model, tumor microenvironment, TCGA

## Abstract

**Background:**

Necroptosis is associated with the development of many tumors but in bladder cancer the tumor microenvironment (TME) and prognosis associated with necroptosis is unclear.

**Methods:**

We classified patients into different necroptosis subtypes by the expression level of NRGS (necroptosis-related genes) and analyzed the relationship between necroptosis subtypes of bladder cancer and TME, then extracted differentially expressed genes (DEGS) of necroptosis subtypes, classified patients into different gene subtypes according to DEGS, and performed univariate COX analysis on DEGS to obtain prognosis-related DEGS. All patients included in the analysis were randomized into the Train and Test groups in a 1:1 ratio, and the prognostic model was obtained using the LASSO algorithm and multivariate COX analysis with the Train group as the sample, and external validation of the model was conducted using the GSE32894.

**Results:**

Two necroptosis subtypes and three gene subtypes were obtained by clustering analysis and the prognosis-related DEGS was subjected to the LASSO algorithm and multivariate COX analysis to determine six predictors to construct the prognostic model using the formula: riskScore = CERCAM × 0.0035 + POLR1H × −0.0294 + KCNJ15 × −0.0172 + GSDMB × −0.0109 + EHBP1 × 0.0295 + TRIM38 × −0.0300. The results of the survival curve, roc curve, and risk curve proved the reliability of the prognostic model by validating the model with the test group and the results of the calibration chart of the Nomogram applicable to the clinic also showed its good accuracy. Necroptosis subtype A with high immune infiltration had a higher risk score than necroptosis subtype B, gene subtype B with low immune infiltration had a lower risk score than gene subtypes A and C, CSC index was negatively correlated with the risk score and drug sensitivity prediction showed that commonly used chemotherapeutic agents were highly sensitive to the high-risk group.

**Conclusion:**

Our analysis of NRGS in bladder cancer reveals their potential role in TME, immunity, and prognosis. These findings may improve our understanding of necroptosis in bladder cancer and provide some reference for predicting prognosis and developing immunotherapies.

## Introduction

In recent years, as research has continued, the perception of the concept of cell death has changed; previously, many believed that cell necrosis was accidental and that it was distinguished from regulated apoptosis, but current research has confirmed that necrosis in specific circumstances may be associated with molecular regulatory mechanisms of intracellular and membrane receptor signaling and this regulated and programmed necrosis is then named necroptosis (1, 2). Necroptosis is a regulatory cell death mediated by RIP 1, RIP 3, and MLKL (3, 4). Many current studies suggest that Necroptosis is associated with many diseases, such as tumors with Parkinson's disease (4–6). Najafov et al. suggest that necrosis promotes tumor metastasis and T-cell death (7). A characteristic of tumor cells is their resistance to the induction of cell death by antitumor agents. In tumorigenic development, Necroptosis activates the antitumor response because of its immunogenic nature and so may provide a direction of treatment to eliminate anti-apoptotic tumor cells (3, 8). Gong et al. suggested that the regulation of Necroptosis-related proteins may be downregulated in colorectal cancer, leukemia, breast cancer, and melanoma, while RIPK1 is upregulated in lung and pancreatic cancer (3). Based on the findings of previous studies, Necroptosis may favor tumor development. Therefore, Necroptosis is a valuable research direction related to the treatment of tumors (3, 8).

Bladder cancer, as one of the common tumors of the urinary system, has a high incidence worldwide and the treatment of bladder cancer has been similar in the past. However, current advances in research on molecular and tumorigenic mechanisms have opened the door to personalized treatment of bladder cancer (9). Furthermore, in studies related to Necroptosis and bladder cancer, Wang et al. found that induction of Necroptosis is another way to overcome apoptosis resistance in BC treatment (10). Yan et al. demonstrated that Troglitazone induced programmed cell death in several types of bladder cancer cells (11) and Cheng et al. showed that ABT-737 inhibited the invasion and proliferation of bladder cancer cells by inducing cell necroptosis (12), and these studies have continuously demonstrated the importance of necroptosis in the treatment of bladder cancer. In the present analysis, we downloaded and integrated gene expression data and clinical information from the TCGA dataset for bladder cancer patients. Cluster analysis was used to explore the differences in TME between subtypes. Then, prognostic features associated with Necroptosis were identified, prediction models were developed, and the validity of the models was further validated. Our results demonstrated that the prognostic model accurately predicted the prognosis of bladder cancer patients and initially revealed the differences in immune microenvironment among different bladder cancer subtypes, providing ideas and insights for predicting prognosis and treatment.

## Materials and Methods

### Data Acquisition

Based on the large amount of data available online, we obtained raw gene expression data and clinically relevant information for bladder cancer from the TCGA database (a total of 410 tumor samples and 19 normal samples. Https://tcga-data.nci.nih.gov/tcga/). In addition, the cohort GSE32894 was obtained from the Gene Expression Overview (Geo) (https://www.ncbi.nlm.nih.gov/geo/) database for subsequent validation. sixty seven necroptosis-related genes (NRG) and their supporting literature were obtained from previous studies (Supplementary Table 1). Background adjustment and quantile normalization were performed on the original files.

### Cluster Analysis of NRGS

Sixty-seven NRGS obtained from previous literature were clustered according to NRGS expression using the “ConsensusClusterPlus” software package and patients were classified into different molecular subtypes. Gene set variation analysis (GSVA) was performed to study the biological differences in NRGS.

### Molecular Subtypes of Bladder Cancer in Relation to Clinical Data, TME

We performed Kaplan-Meier analysis of the different molecular subtypes of bladder cancer to show the differences in median OS between them and to compare the molecular subtypes with the clinical situation. We calculated stromal and immune scores of the patients, which were obtained based on the ESTIMATE algorithm. Single sample gene set enrichment analysis (SsGSEA) was used to investigate differences in immune cell content between molecular subtypes of bladder cancer.

### Acquisition of DEGS in Molecular Subtypes of Bladder Cancer and Its Functional Analysis

The DEGS of different Necroptosis subtypes were extracted by “limma” (adjusted *p* < 0.001 and fold change >4) and, after obtaining the Necroptosis-related DEGS, we performed GO and KEGG to investigate their potential functions.

### Necroptosis-Related Prognostic Modeling, Nomogram Suitable for Individualized Treatment

On the one hand, we performed univariate Cox analysis of DEGS between different Necroptosis subtypes to obtain DEGS associated with prognosis while, on the other hand, cluster analysis was performed on patients according to DEGS between Necroptosis subtypes to classify patients into different subtype groups (i.e., gene subtypes). Finally, all patients included in the analysis were randomly and equally divided into a Train group (201 samples) and Test group (201 samples) and ALL group represented all sample groups. On the basis of Train group as samples, the minimum optimal results were obtained using Lasso algorithm and multivariate COX analysis was performed to obtain the model and then the model was validated with the Test group (The median risk score of Train group was used to classify patients into high and low risk groups) and a new nomogram for bladder cancer patients was created.

### Immunoreactivity Between High and Low Risk Groups, Correlation of Tumor Stem Cells With Risk Scores

We performed immunoreactivity analyses between the two subgroups distinguished by the model to obtain differences in immune cell content and immune pathways, in addition to assessing the relationship between risk scores and tumor stem cells.

### Drug Sensitivity Analysis

We carried out sensitivity prediction of drugs for commonly used chemotherapeutic agents in both risk groups to investigate the differences in the sensitivity of chemotherapeutic agents between them. We used the pRRophetic software package to calculate the semi-inhibitory concentration (IC50) values of commonly used chemotherapeutic agents, where the higher the IC50 value, the lower the drug sensitivity.

### Statistical Analysis

All statistical analyses were performed using the perl software and R software (version 4.1.2), packages: limma, ggplot2, pheatmap, DOSE, clusterProfiler, igraph, Rtsne, glmnet, survminer, survival, org.Hs.eg.db, timeROC, caret, pRRophetic, ggpubr, GSVA, GSEABase, ggExtra, preprocessCore, enrichplot, ConsensusClusterPlus, maftools, rms, regplot, RCircos, ggalluvial, psych, estimate *P*-value < 0.05 for all analyses in this study, if not otherwise stated.

In all figures: “^*^” = 0.05, “^**^” = 0.01, “^***^” = 0.001.

## Results

### Alteration of NRGS in Bladder Cancer

**Figure 1:**
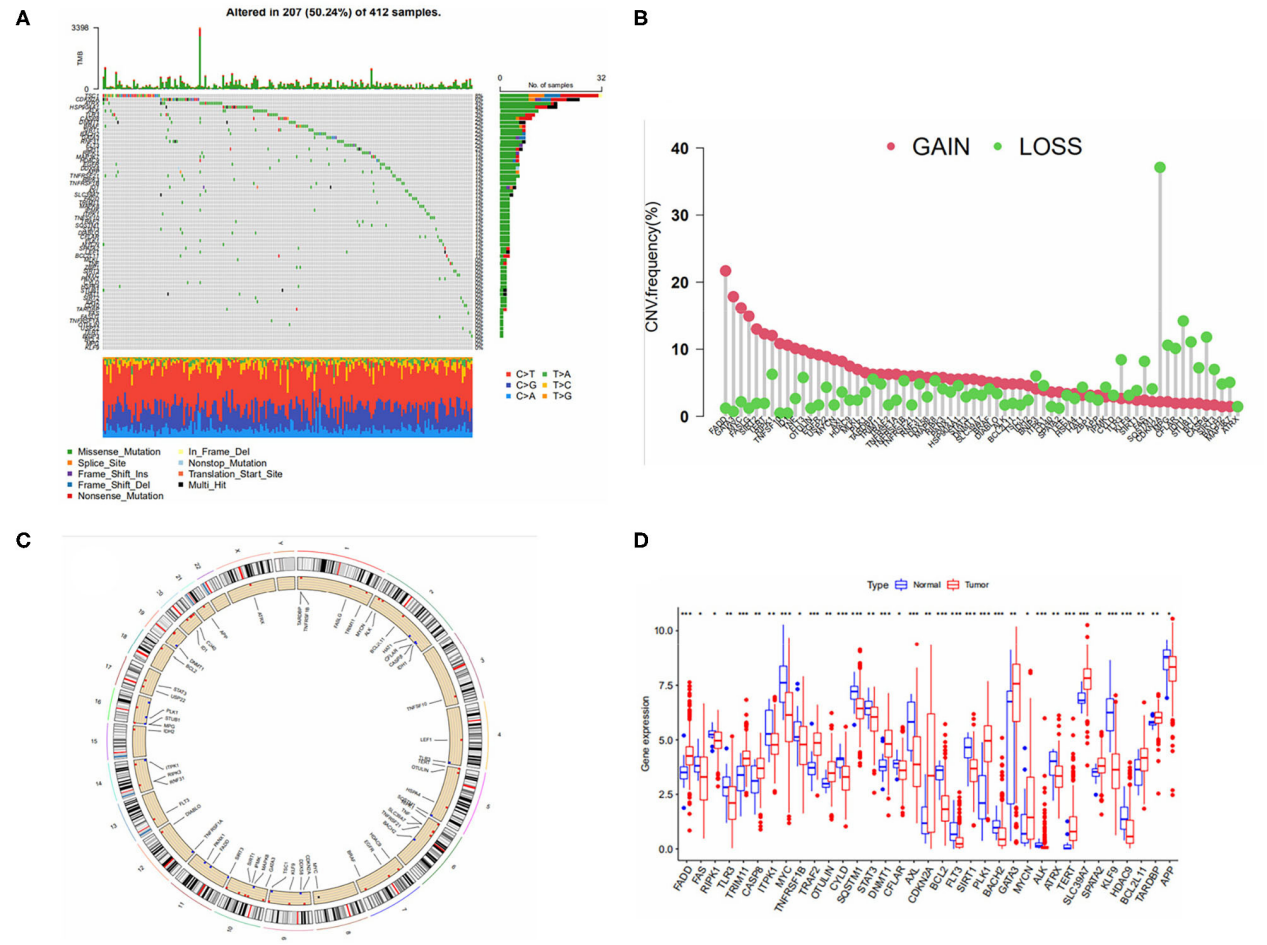
**(A)** Mutation frequency of 67 necroptosis -related genes in 412 bladder cancer patients from the TCGA cohort. **(B)** Frequencies of CNV gain, loss, and non-CNV among NRGs. **(C)** Location of CNV in NRGS on 24 chromosomes. **(D)** Expression distributions of 67 NRGs between normal and tumor tissues.

A total of 67 Necroptosis-related genes (NRGS) were included in this study. Mutation data from the TCGA-BLCA cohort showed that more than 50 percent of the 412 samples had mutations in NRGS (Figure 1A). We analyzed the somatic copy number of NRGS, and the results in Figure 1B show that all 67 NRGS had CNV alterations, of which CDKN2A has nearly 40% of the copy number variation loss, and the rest of the NGRS have varying degrees of copy number variation increase or deletion. Figure 1C represents the chromosomal alterations of CNV in NRGS. Figure 1D shows the difference in gene expression levels between bladder cancer samples and normal samples. Interestingly we found that CDKN2A,FADD,GATA3,MYC were highly variable between gain and loss in CNV, while at the mRNA expression level these same NRGS were also found to be significantly different in normal tissues compared to tumor tissues. We speculate that CNV may regulate mRNA expression levels, but of course, CNV is not the only regulator, and these results suggest a potential role of NRGS in bladder cancer.

### Necroptosis-Associated Subtypes of Bladder Cancer

**Figure 2:**
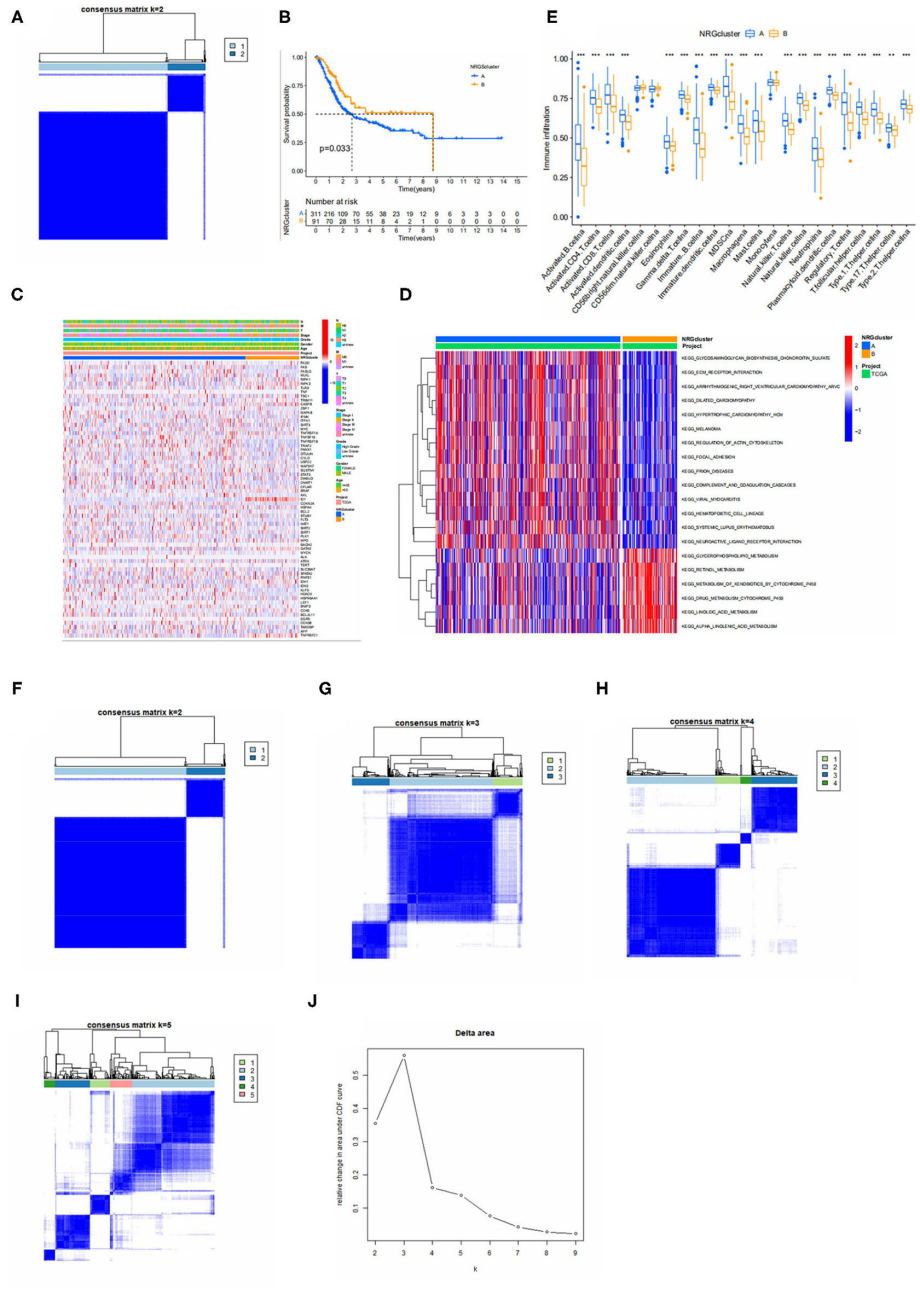
**(A)** Consensus matrix heatmap defining two clusters (*k* = 2) and their correlation area. **(B)** Kaplan-Meier analysis curves of NRGS subtypes. **(C)** Differences in clinicopathologic features and expression levels of NRGs between the two distinct subtypes. **(D)** GSVA of biological pathways between two distinct subtypes, in which red and blue represent activated and blue inhibited pathways, respectively. **(E)** Relative content of 23 immune cells in different Necroptosis subtypes. **(F–I)** Defined as two, three, four and five subtypes (*k* = 2, 3, 4, and 5) and their areas. **(J)** Cumulative distribution function curve. The figure shows a consistent cumulative distribution function plot for each type of clustering (different *k*) to determine the choice of k when the CDF is maximized so that consistency and clustering confidence are maximized.

To further recognize the expression characteristics of NRGS in bladder cancer, we classified patients using 67 NRGS expression levels as the basis for clustering analysis. The results are shown in Figure 2A, where the patients were classified into two subtypes A, B (*K* = 2). From Figures 2F–J, *k* = 2 seems to be the most appropriate. Then a Kaplan-Meier analysis was performed on the two subtypes, and the results (Figure 2B) showed that the median overall survival rate between them were differential (*p* < 0.05). In addition, NRG subtypes and clinically relevant information were compared (Figure 2C).

Continuing the GSVA analysis of the two NRGS-related subtypes mentioned above, it can be found that the first 14 pathways are more significantly enriched in subtype A, including melanoma, extracellular matrix receptor interactions (Pupa et al. found that degradation of the extracellular matrix is a prerequisite for metastatic invasion of tumor cells), neuroactive ligand-receptor interactions, etc., and the latter 6 pathways are intracellular certain substance metabolic pathways, which were significantly enriched on the B subtype (Figure 2D). To further explore the situation of NRGS in TME of bladder cancer, we performed an immune cell correlation analysis with a total of 23 immune cells and it was observed that there was a significant difference between subtypes A and B in 20 immune cell infiltrations (Figure 2E), and all immune cells of subtype A were higher than those of subtype B.

### Gene Subtypes Based on Differential Genes

**Figure 3:**
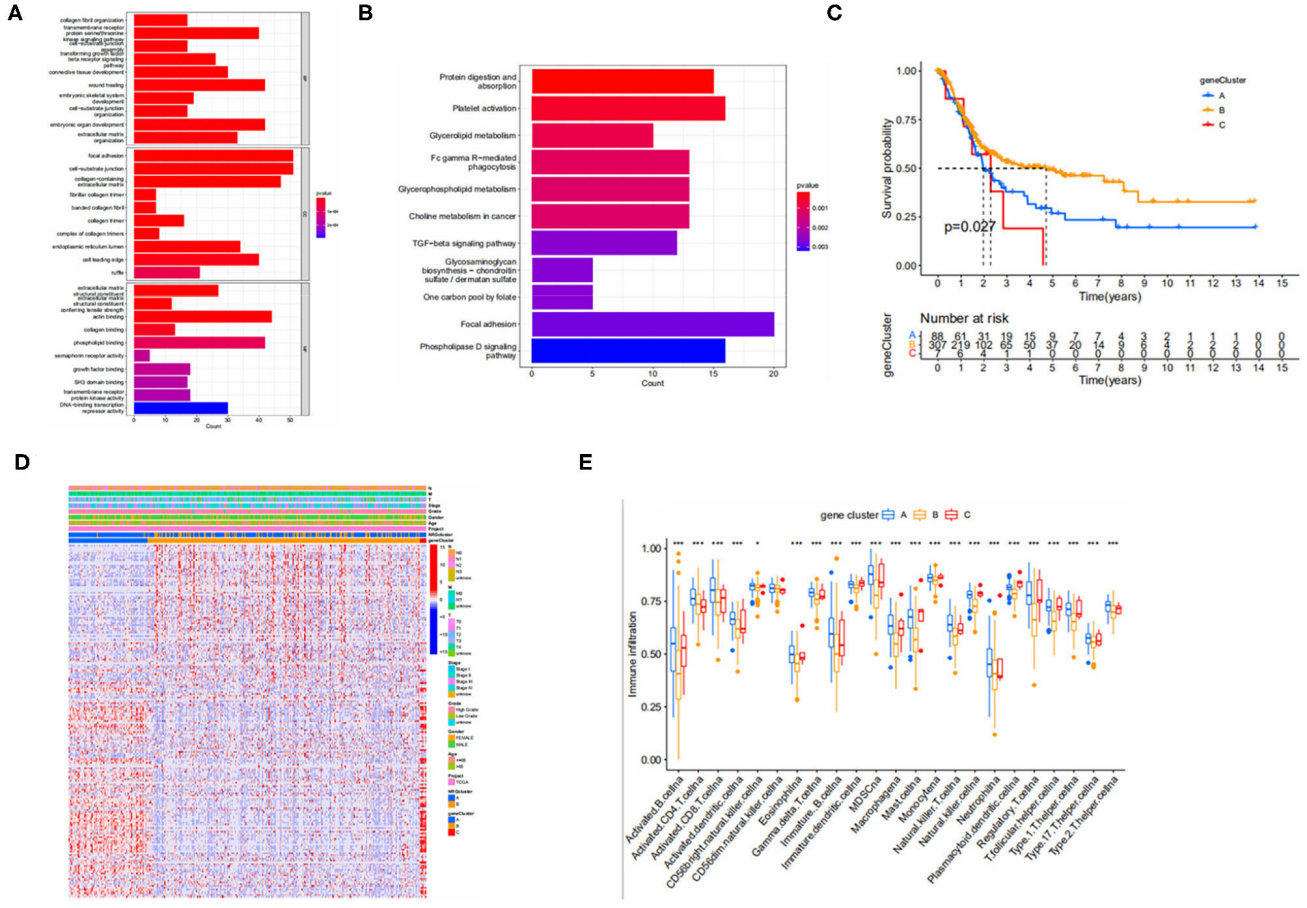
**(A,B)** GO and KEGG enrichment analyses of DEGs among two necroptosis-associated subtypes. (GO, Gene Ontology; KEGG, Kyoto Encyclopedia of Genes and Genomes). **(C)** Kaplan-Meier analysis curves for the three gene subtypes. **(D)** Relationships between clinicopathologic features and the three gene subtypes. **(E)** Immune infiltration between gene subtypes.

To further explore the potential biological behavior among NRGS subtypes we obtained 1,063 DEGS associated with necroptosis subtypes using limma and GO (Figure 3A) and kegg (Figure 3B) were performed on these genes. GO results showed an association with extracellular matrix, and degradation of extracellular matrix is a prerequisite for tumor cell metastasis and invasion (13). Choline metabolism in cancer was observed in the KEGG results, suggesting some potential role of necroptosis in tumors. Next, we performed univariate COX analysis of DEGS to obtain 195 prognosis-related DEGS. Based on these 195 prognosis-related DEGS, we performed consensus clustering analysis of patients to obtain three gene subtypes (type A, type B, and type C) and Kaplan-Meier analysis of the three gene subtypes (Figure 3C). It can be seen that gene subtype A has the highest median OS. We also visualized the genotyping in relation to the clinical profile (Figure 3D). The results of immune infiltration show us that genotype A has a higher degree of immune infiltration than the remaining two subtypes (Figure 3E).

### Necroptosis-Related Model Construction and Validation

We performed lasso and multivariate COX analysis on the basis of prognosis-related DEGS with the Train group (201 specimens) as the sample. Lasso algorithm was performed in order to obtain the minimum best fit and multivariate COX analysis further filtered out six genes to construct the model, expressed by the formula: riskScore = CERCAM × 0.0035 + POLR1H × −0.0294 + KCNJ15 × −0.0172 + GSDMB × −0.0109 + EHBP1 × 0.0295 + TRIM38 × −0.0300, where CERCAM and EHBP1 are high-risk genes, and KCNJ15, GSDMB, TRIM38 and POLR1H are low-risk genes.

**Figure 4:**
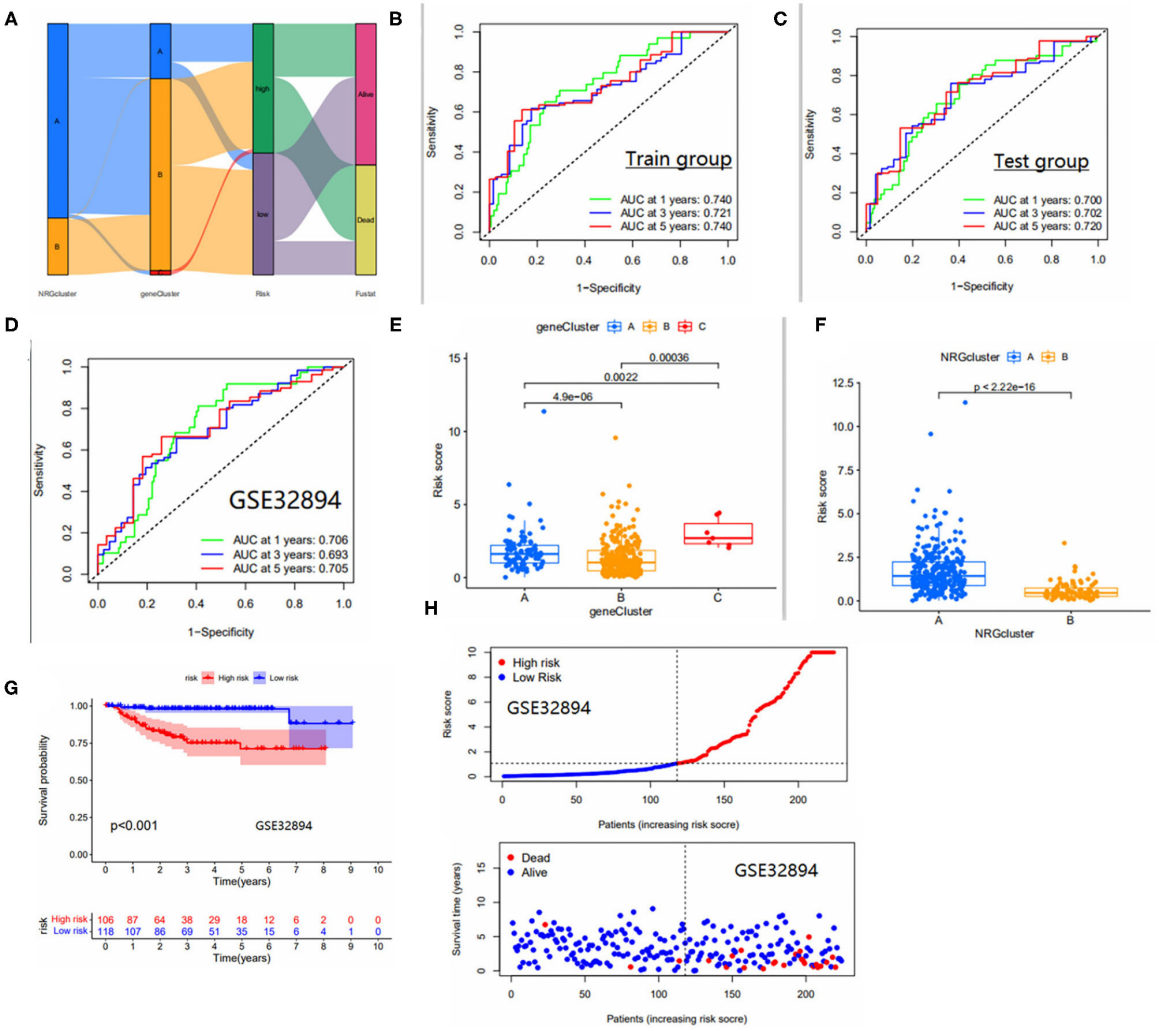
**(A)** Distribution of the two NRGS subtypes and three genetic subtypes between high and low risk groups. **(B–D)** Area under the ROC curve for the Train group/test group, GSE32894 cohort. ROC curves to predict the sensitivity and specificity of 1-, 3-, and 5-year survival according to the risk_score. **(E)** Differences in risk scores between gene subtypes. **(F)** Differences in risk scores between NRGS subtypes. **(G)** Survival curves for high and low risk groups in the GSE32894 cohort. **(H)** Risk curves for high and low risk groups in the GSE32894 cohort. Ranked dot and scatter plots showing the risk_score distribution and patient survival status.

We show the distribution of patients in two risk score groups, two necroptosis subtypes, and three genetic subtypes in the form of a sankey diagram (Figure 4A). The risk scores were significantly different between subtypes and the risk score for necroptosis subtype A was significantly higher than that for necroptosis subtype B (Figure 4F) and, from the previous immune correlation analysis, we concluded that the degree of immune cell infiltration was higher in all subtypes A than in subtype B. Therefore, we speculate that the risk score may be closely related to immune infiltration. The risk score for genotype C was significantly higher than the remaining two groups (Figure 4E). In fact, this finding can also be inferred from the overall survival rate of the patients, because the survival time of patients with genotype C was generally shorter, which laterally reflects that the risk score is a reliable predictor of patient survival. To observe the validity of the model, we validated the model internally and added the dataset GSE32894 for external validation.

**Figure 5:**
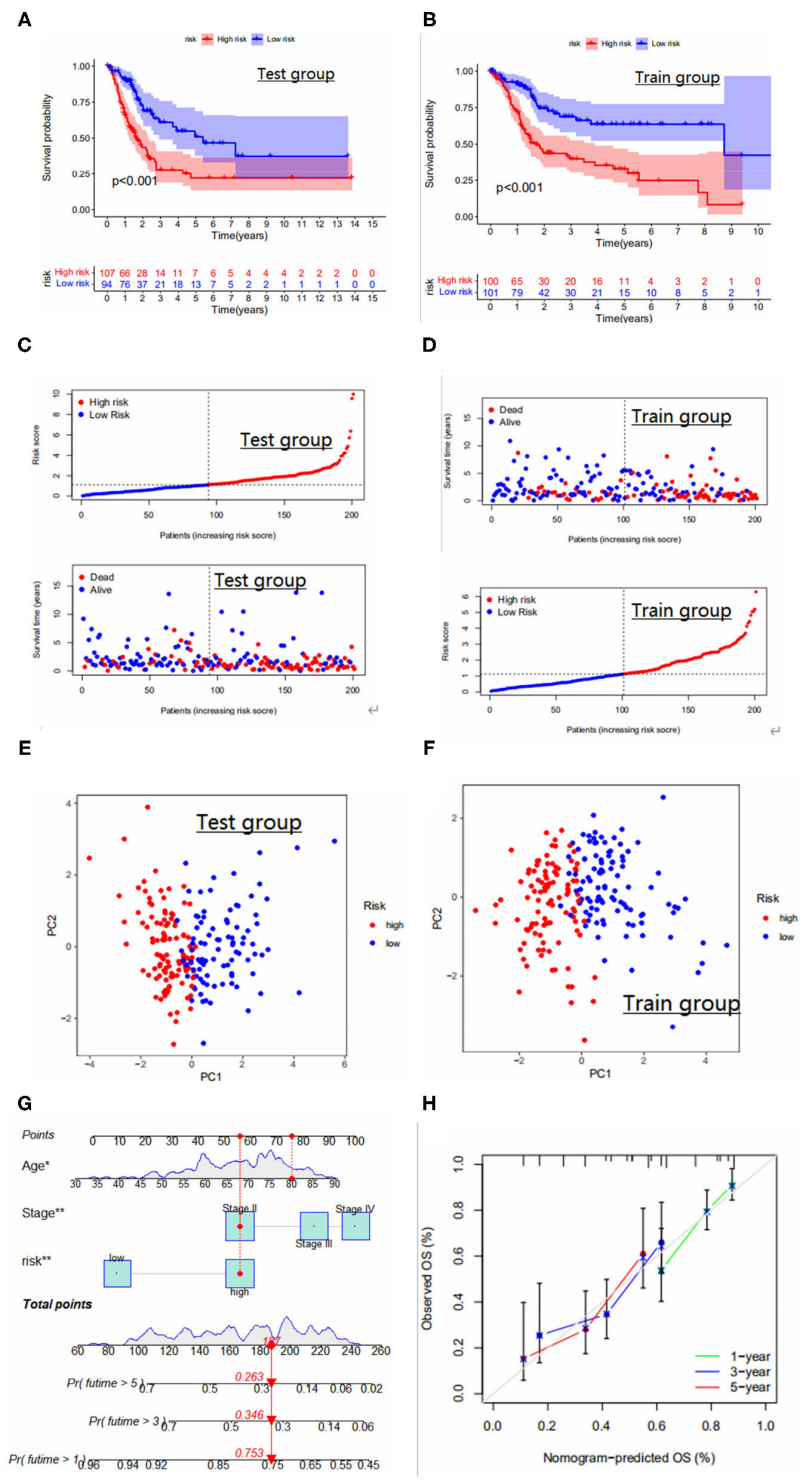
**(A,B)** Survival curves for high and low risk groups in the Test and Train groups. **(C,D)** Risk curves for high and low risk groups in the Test and Train groups. Ranked dot and scatter plots showing the risk_score distribution and patient survival status. **(E,F)** PCA results for the Test and Train groups. PCA analysis based on the prognostic signature. The high and low-risk patients are represented by red and steel blue dots, respectively. **(G)** Nomogram for predicting 1-year, 3-year, and 5-year survival rates for bladder cancer patients. **(H)** Calibration curves of nomogram for predicting survival in bladder cancer patients in the test group.

The area under the ROC curve was higher than 0.65 in both internal validation and external validation (Figures 4B–D), suggesting good predictive ability of the model. The Kaplan-Meier analysis and risk curve results for the GEO group are shown in Figures 4G,H. Kaplan-Meier analysis of the train and test groups showed that the high-risk group predicted a poorer prognosis for patients with bladder cancer (Figures 5A,B). We observed the correlation between individual risk scores and individual survival in bladder cancer patients from the results of risk curves (Figures 5C,D) and we found that most of the patients who died were in the high-risk group, thus presumably indicating decreasing survival as the risk score increased. Then, we performed PCA and the results showed that we could distinguish well-between high-risk and low-risk patients (Figures 5E,F), which also illustrates the excellent ability of the model to distinguish between high- and low-risk. Finally, to make the model easy to use in the clinical setting, we created a new nomogram (Figure 5G) and the calibration plots showed that the predictive accuracy of the nomogram was better in the test groups (Figure 5H). The risk score obtained from the cox model allows us to distinguish patients into high and low risk groups, and then patients are further assigned the corresponding scores in the nomogram, and the remaining predictors (age and tumor stage) are also assigned their respective scores. Figure 5G shows the 16th patient in the test dataset, 80 years old, with a risk score of 1.3234; the patient belongs to the high-risk group and at stage ii tumor stage. As can be observed in the figure, the patient's scores were 76,55.5 and 55.5, respectively, with final overall scores of 187.1-, 3- and 5-year survival rates of 0.753, 0.346 and 0.263, respectively.

### TME Between Two Risk Groups, and the Relationship With CSC Index

**Figure 6:**
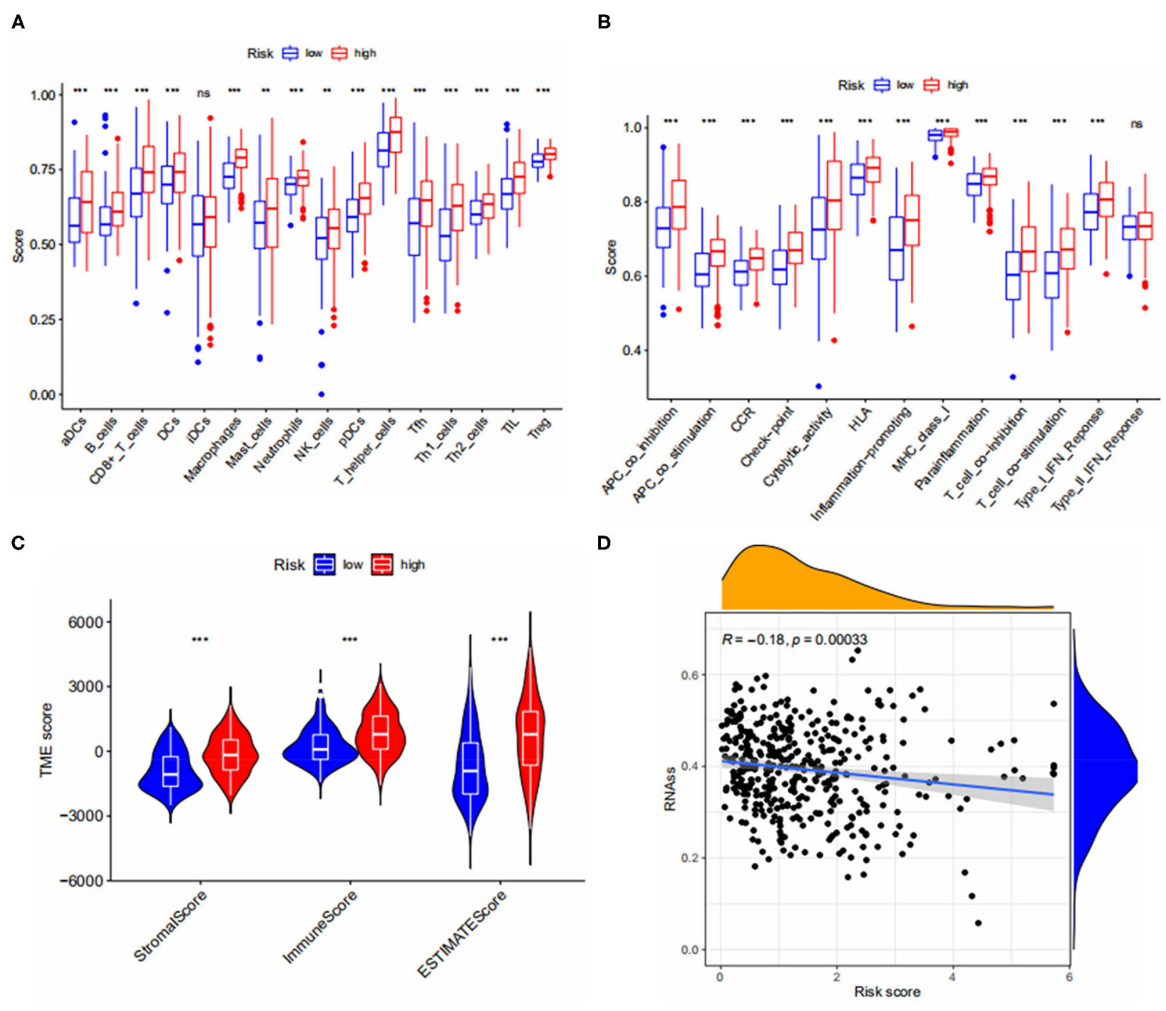
**(A,B)** Immune cell infiltration and immune pathway activity scores in both risk groups. **(C)** Correlations between risk groups and both immune and stromal scores. **(D)** Relationship between CSC Index and risk score. *** < 0.001.

We calculated immune cell infiltration scores for both risk groups and assessed the activity of immune pathways. Fifteen of 16 immune cells and 12 of 13 immune-related pathways were significantly different between the two subgroups and we found that the degree of immune infiltration and the activity of immune pathways were all higher in the high risk group than in the low risk group (Figures 6A,B) and, on further assessing the differences in TME between the two risk groups, the results are shown in Figure 6C, StromalScore, ImmuneScore and ESTIMATEScore were significantly different between the two risk subgroups and the TME score in the high-risk group was higher in StromalScore, ImmuneScore, and ESTIMATEScore were all higher in the high-risk group than in the low-risk group, which is consistent with our hypothesis. Finally, we calculated the relationship between risk score and CSC index, and the results showed that bladder cancer cells with lower risk scores had a lower degree of cell differentiation and more abundant stem cell properties (Figure 6D).

### Drug Sensitivity

**Figure 7:**
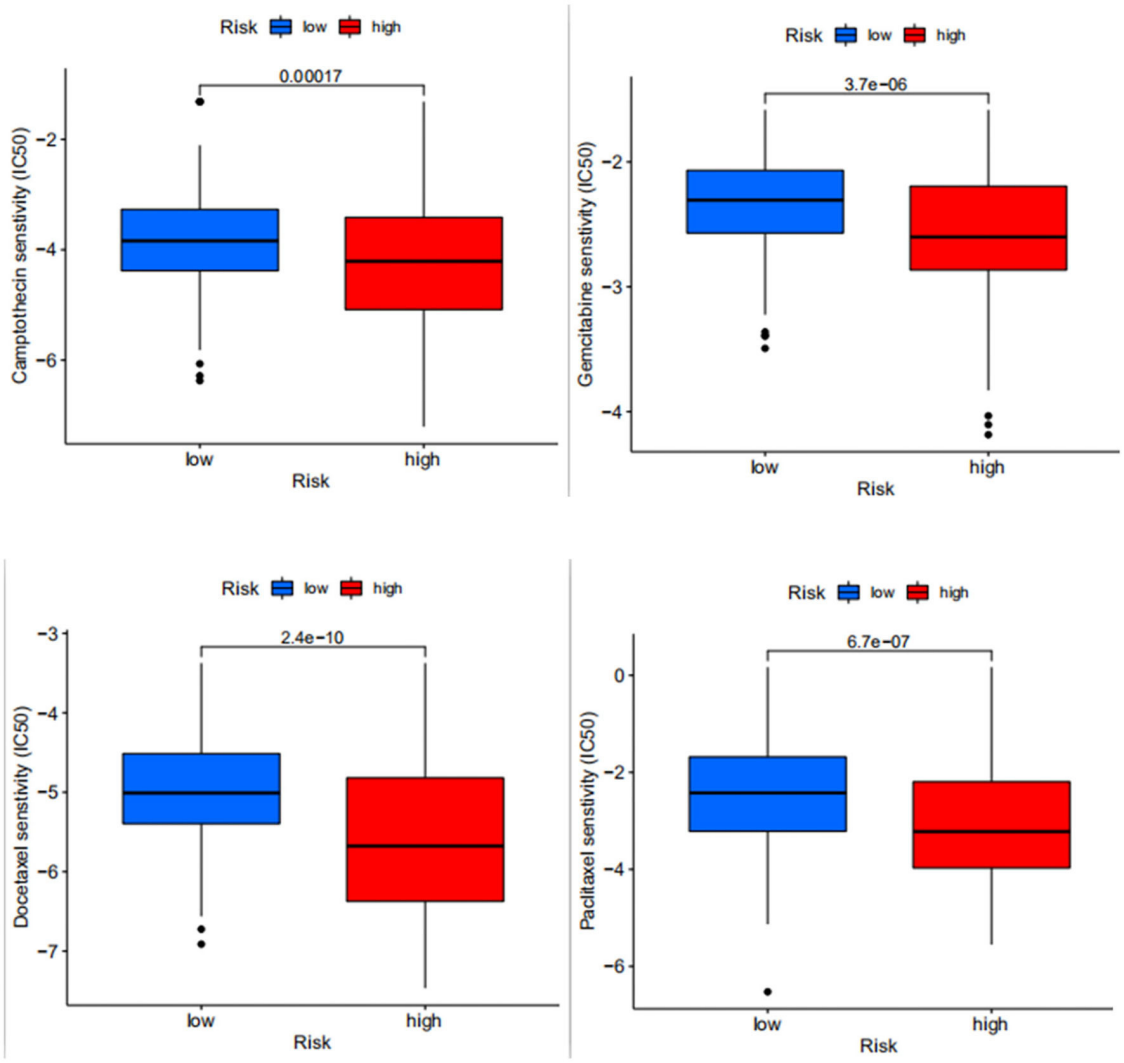
Differences in drug sensitivity between docetaxel, gemcitabine, paclitaxel and camptothecin in high- and low-risk patients, where the higher the IC50 value of the drug, the lower the sensitivity.

We predicted the drug sensitivity of some common chemotherapeutic agents for bladder cancer, such as docetaxel, gemcitabine, paclitaxel, and camptothecin (Figure 7) and among these common chemotherapeutic agents, it was seen that the sensitivity was higher in the high-risk group than in the low-risk group. This suggests that the high-risk patients distinguished by the model are more sensitive to common chemotherapeutic agents, so when we screen high-risk patients with the model, can we prioritize chemotherapy for high-risk patients if the overall situation allows?

## Discussion

Necroptosis has a bidirectional role in tumor development and may promote or inhibit the progression of tumor cells (14, 15). Ando et al. concluded that necroptosis plays a crucial role in the invasion and migration of many tumors (16), making cancer cell Necroptosis a promising anti-cancer approach (17). There is evidence that the tumor microenvironment has a significant impact in tumor treatment, progression, and drug resistance (18). Scarpitta et al. (19) suggested that induction of cellular necroptosis, which constitutes the tumor microenvironment, may be able to link immunosuppressive TME and thus reduce tumor cell resistance to antitumor therapies. Molecular subtypes are associated with tumor immunosuppression and microenvironment (20, 21). Thus, in our study, we described the expression and its mutation of NRGS in bladder cancer patients, initially explored the impact of necroptosis in bladder cancer, and distinguished different necroptosis subtypes based on the expression of NRGS. Our analysis showed that subtype A had a higher level of immune infiltration and a higher risk score than subtype B. In contrast, subtype B not only had a lower level of immune infiltration than subtype A but also had a lower risk score, suggesting a potential role of NRGS in bladder cancer and an impact on TME. Gene subtypes distinguished by prognostic DEGS expression also differed significantly in terms of degree of immune infiltration and risk score; the degree of immune infiltration and risk scores for genotype B were lower than the remaining two subtypes. Among the gene subtypes, the results of Kaplan-Meier analysis of gene subtype B showed its good median os and also demonstrated its low risk score compared to the other two subtypes in our analysis, thus one can attempt to predict a good prognosis in people conforming to gene subtype B. Similarly, gene subtype C may also attempt to predict poor prognosis in patients with bladder cancer and their relatively high immune infiltration status may benefit from immunotherapy.

In recent years, immunotherapy has been applied to treat many cancers, but immunotherapy is not a panacea (22) and immunosuppressive TME has led to a number of patients not benefiting from immunotherapy, and the classification of tumors based on immune correlation is now given the name hot and cold tumors. The difference between hot and cold tumors is the degree of immune scoring, with hot tumors benefiting from multiple immunotherapies and cold tumors failing to release a low degree of T-cell immune function during immunotherapy resulting in poor therapeutic outcomes (23, 24), Necroptosis subtype A and gene subtype A with high immune infiltration can be considered to some extent as hot tumors and benefit in immunotherapy. Of course, both TME and immune scoring are different in different subtypes, which may also lead to different prognostic and immunotherapeutic outcomes (25, 26). Therefore, in this study, we interconnected the necroptosis subtype, the gene subtype and the prognostic model, hoping to describe them in the TME. The characteristics of each immune cell in the tumor microenvironment can predict clinical outcome to varying degrees, for example, the high infiltration of Tregs suppresses the anti-cancer immune response and leads to a poor prognosis (27). However, this needs to be judged on a comprehensive basis as prognostic factors affecting tumors are diverse and may be interrelated. Therefore, it is promising to use our analysis on TME and construct effective prognostic models for patient prognosis, immunotherapy.

We constructed a prognostic model based on Necroptosis subtype-related prognosis DEGS by lasso algorithm and multivariate COX analysis, which consists of 6 predictors (CERCAM POLR1H, KCNJ15, GSDMB, EHBP1, TRIM38), among which CERCAM is closely related to bladder cancer. Zuo et al. (28) demonstrated that *in vitro*, CERCAM overexpression significantly promoted bladder cancer cell survival, cell invasion and DNA synthesis, altering the expression patterns of N-cadherin, E-cadherin and Caspase 3; *in vivo*, CERCAM silencing inhibited tumor progression in mice. He et al. found that GSDMB could bind to STAT 3, thereby activating STAT 3 signaling in bladder cancer, and demonstrated that GSDM could interact with USP 24B to hinder the degradation of GSDMB in bladder cancer, thus the USP 24/GSDMB/STAT 3 axis could be a new signaling pathway for targeted bladder cancer therapy (29). Wang et al. demonstrated that TRIM 38 plays an inhibitory role in the development of bladder cancer, and the TRIM 38/GLUT 1 axis is of great importance as a weak point for bladder cancer treatment (30). The relationship between the remaining three predictors and bladder cancer is not yet supported by relevant experimental evidence and literature; therefore, they may provide options for later investigators when studying potential therapeutic targets for bladder cancer.

The shortcoming of our study is that the remaining three predictors need experimental validation and, of course, the selection bias of the public database sample contributes to some limitations of this study. Finally, the results of risk scores may be influenced due to complex interactions between genes. Further learning of new modeling approaches, such as ensemble modeling, is worthwhile in future studies (31).

## Conclusion

Our correlation analysis of NRGS explored the regulatory mechanisms affecting tumor-TME-immunotherapy-prognosis. On this basis, we constructed a prognostic model for bladder cancer and a new nomogram to predict patient survival. These findings suggest a role of necroptosis in bladder cancer and suggest six potential therapeutic targets for bladder cancer, which also provide a reference for individualized immunotherapy for bladder cancer patients.

## Data Availability Statement

The original contributions presented in the study are included in the article/Supplementary Materials, further inquiries can be directed to the corresponding author/s.

## Author Contributions

SN conceived and drafted the manuscript. YH and JH assisted with statistics. YH organized the data. SK and FC revised the final manuscript. All authors read and approved the final manuscript.

## Conflict of Interest

The authors declare that the research was conducted in the absence of any commercial or financial relationships that could be construed as a potential conflict of interest.

## Publisher's Note

All claims expressed in this article are solely those of the authors and do not necessarily represent those of their affiliated organizations, or those of the publisher, the editors and the reviewers. Any product that may be evaluated in this article, or claim that may be made by its manufacturer, is not guaranteed or endorsed by the publisher.

## Supplementary Material

Supplementary Table 1 67 Necroptosis-related genes (NRGS) and their supporting literature were obtained from previous research reports.

## Abbreviations

NRGS: Necroptosis -related genes
DEGs: Differentially expressed genes
TCGA: The genomic data commons data portal and the cancer genome atlas database
ROC curve: Receiver operating characteristic curve
LASSO: the least absolute shrinkage and selection operator Cox regression algorithm
PCA: principal component analysis
CNV: copy number variation
CSC: cancer stem cell
GSVA: Gene set variation analysis
SsGSEA: single-sample gene set enrichment analysis
OS: Overall survival
GO: Gene Ontology
KEGG: Kyoto Encyclopedia of Genes and Genomes
TME: Tumor microenvironment.
