# Supplementary material for: Identification of Bladder Cancer Subtypes Based on Necroptosis-Related Genes, Construction of a Prognostic Model
Source: Front Surg. 2022 Apr 11;9:860857. doi: 10.3389/fsurg.2022.860857 (PMC9035642; doi:10.3389/fsurg.2022.860857)
Supplement: Supplementary Table 1 — 67 Necroptosis-related genes (NRGS) and their supporting literature were obtained from previous research reports. [file Table_1.DOCX]

Table S1

| Genes | Source |
| --- | --- |
| FADD | necroptosis geneset M24779.gmt |
| FAS | necroptosis geneset M24779.gmt |
| FASLG | necroptosis geneset M24779.gmt |
| MLKL | necroptosis geneset M24779.gmt |
| RIPK1 | necroptosis geneset M24779.gmt |
| RIPK3 | necroptosis geneset M24779.gmt |
| TLR3 | necroptosis geneset M24779.gmt |
| TNF | necroptosis geneset M24779.gmt |
| TSC1 | Xie Y, Zhao Y, Shi L, Li W, Chen K, Li M, Chen X, Zhang H, Li T, Matsuzawa-Ishimoto Y, Yao X, Shao D, Ke Z, Li J, Chen Y, Zhang X, Cui J, Cui S, Leng Q, Cadwell K, Li X, Wei H, Zhang H, Li H, Xiao H. Gut epithelial TSC1/mTOR controls RIPK3-dependent necroptosis in intestinal inflammation and cancer. J Clin Invest. 2020 Apr 1;130(4):2111-2128. doi: 10.1172/JCI133264. PMID: 31961824; PMCID: PMC7108921. |
| TRIM11 | Xie Y, Zhao Y, Shi L, Li W, Chen K, Li M, Chen X, Zhang H, Li T, Matsuzawa-Ishimoto Y, Yao X, Shao D, Ke Z, Li J, Chen Y, Zhang X, Cui J, Cui S, Leng Q, Cadwell K, Li X, Wei H, Zhang H, Li H, Xiao H. Gut epithelial TSC1/mTOR controls RIPK3-dependent necroptosis in intestinal inflammation and cancer. J Clin Invest. 2020 Apr 1;130(4):2111-2128. doi: 10.1172/JCI133264. PMID: 31961824; PMCID: PMC7108921. |
| CASP8 | Fritsch M, Günther SD, Schwarzer R, Albert MC, Schorn F, Werthenbach JP, Schiffmann LM, Stair N, Stocks H, Seeger JM, Lamkanfi M, Krönke M, Pasparakis M, Kashkar H. Caspase-8 is the molecular switch for apoptosis, necroptosis and pyroptosis. Nature. 2019 Nov;575(7784):683-687. doi: 10.1038/s41586-019-1770-6. Epub 2019 Nov 20. PMID: 31748744. |
| ZBP1 | Yang D, Liang Y, Zhao S, Ding Y, Zhuang Q, Shi Q, Ai T, Wu SQ, Han J. ZBP1 mediates interferon-induced necroptosis. Cell Mol Immunol. 2020 Apr;17(4):356-368. doi: 10.1038/s41423-019-0237-x. Epub 2019 May 10. PMID: 31076724; PMCID: PMC7109092. |
| MAPK8 | Wang Y, Zhao M, He S, Luo Y, Zhao Y, Cheng J, Gong Y, Xie J, Wang Y, Hu B, Tian L, Liu X, Li C, Huang Q. Necroptosis regulates tumor repopulation after radiotherapy via RIP1/RIP3/MLKL/JNK/IL8 pathway. J Exp Clin Cancer Res. 2019 Nov 9;38(1):461. doi: 10.1186/s13046-019-1423-5. PMID: 31706322; PMCID: PMC6842489. |
| IPMK | Dovey CM, Diep J, Clarke BP, Hale AT, McNamara DE, Guo H, Brown NW Jr, Cao JY, Grace CR, Gough PJ, Bertin J, Dixon SJ, Fiedler D, Mocarski ES, Kaiser WJ, Moldoveanu T, York JD, Carette JE. MLKL Requires the Inositol Phosphate Code to Execute Necroptosis. Mol Cell. 2018 Jun 7;70(5):936-948.e7. doi: 10.1016/j.molcel.2018.05.010. Epub 2018 Jun 7. PMID: 29883610; PMCID: PMC5994928. |
| ITPK1 | Dovey CM, Diep J, Clarke BP, Hale AT, McNamara DE, Guo H, Brown NW Jr, Cao JY, Grace CR, Gough PJ, Bertin J, Dixon SJ, Fiedler D, Mocarski ES, Kaiser WJ, Moldoveanu T, York JD, Carette JE. MLKL Requires the Inositol Phosphate Code to Execute Necroptosis. Mol Cell. 2018 Jun 7;70(5):936-948.e7. doi: 10.1016/j.molcel.2018.05.010. Epub 2018 Jun 7. PMID: 29883610; PMCID: PMC5994928. |
| SIRT3 | Tang X, Li Y, Liu L, Guo R, Zhang P, Zhang Y, Zhang Y, Zhao J, Su J, Sun L, Liu Y. Sirtuin 3 induces apoptosis and necroptosis by regulating mutant p53 expression in small‑cell lung cancer. Oncol Rep. 2020 Feb;43(2):591-600. doi: 10.3892/or.2019.7439. Epub 2019 Dec 17. PMID: 31894331. |
| MYC | Seong D, Jeong M, Seo J, Lee JY, Hwang CH, Shin HC, Shin JY, Nam YW, Jo JY, Lee H, Kim HJ, Kim HR, Oh JH, Ha SJ, Kim SJ, Roe JS, Kim W, Cheong JW, Bae KH, Lee SC, Oberst A, Vandenabeele P, Shin DH, Lee EW, Song J. Identification of MYC as an antinecroptotic protein that stifles RIPK1-RIPK3 complex formation. Proc Natl Acad Sci U S A. 2020 Aug 18;117(33):19982-19993. doi: 10.1073/pnas.2000979117. Epub 2020 Aug 4. PMID: 32753382; PMCID: PMC7443878. |
| TNFRSF1A | Seifert L, Werba G, Tiwari S, Giao Ly NN, Alothman S, Alqunaibit D, Avanzi A, Barilla R, Daley D, Greco SH, Torres-Hernandez A, Pergamo M, Ochi A, Zambirinis CP, Pansari M, Rendon M, Tippens D, Hundeyin M, Mani VR, Hajdu C, Engle D, Miller G. The necrosome promotes pancreatic oncogenesis via CXCL1 and Mincle-induced immune suppression. Nature. 2016 Apr 14;532(7598):245-9. doi: 10.1038/nature17403. Epub 2016 Apr 6. Erratum in: Nature. 2021 Mar;591(7851):E28. PMID: 27049944; PMCID: PMC4833566. |
| TNFSF10 | Seifert L, Werba G, Tiwari S, Giao Ly NN, Alothman S, Alqunaibit D, Avanzi A, Barilla R, Daley D, Greco SH, Torres-Hernandez A, Pergamo M, Ochi A, Zambirinis CP, Pansari M, Rendon M, Tippens D, Hundeyin M, Mani VR, Hajdu C, Engle D, Miller G. The necrosome promotes pancreatic oncogenesis via CXCL1 and Mincle-induced immune suppression. Nature. 2016 Apr 14;532(7598):245-9. doi: 10.1038/nature17403. Epub 2016 Apr 6. Erratum in: Nature. 2021 Mar;591(7851):E28. PMID: 27049944; PMCID: PMC4833566. |
| TNFRSF1B | Borghi A, Verstrepen L, Beyaert R. TRAF2 multitasking in TNF receptor-induced signaling to NF-κB, MAP kinases and cell death. Biochem Pharmacol. 2016 Sep 15;116:1-10. doi: 10.1016/j.bcp.2016.03.009. Epub 2016 Mar 16. PMID: 26993379. |
| TRAF2 | Borghi A, Verstrepen L, Beyaert R. TRAF2 multitasking in TNF receptor-induced signaling to NF-κB, MAP kinases and cell death. Biochem Pharmacol. 2016 Sep 15;116:1-10. doi: 10.1016/j.bcp.2016.03.009. Epub 2016 Mar 16. PMID: 26993379. |
| PANX1 | Douanne T, André-Grégoire G, Trillet K, Thys A, Papin A, Feyeux M, Hulin P, Chiron D, Gavard J, Bidère N. Pannexin-1 limits the production of proinflammatory cytokines during necroptosis. EMBO Rep. 2019 Oct 4;20(10):e47840. doi: 10.15252/embr.201947840. Epub 2019 Aug 14. PMID: 31410978; PMCID: PMC6776911. |
| OTULIN | Heger K, Wickliffe KE, Ndoja A, Zhang J, Murthy A, Dugger DL, Maltzman A, de Sousa E Melo F, Hung J, Zeng Y, Verschueren E, Kirkpatrick DS, Vucic D, Lee WP, Roose-Girma M, Newman RJ, Warming S, Hsiao YC, Kőműves LG, Webster JD, Newton K, Dixit VM. OTULIN limits cell death and inflammation by deubiquitinating LUBAC. Nature. 2018 Jul;559(7712):120-124. doi: 10.1038/s41586-018-0256-2. Epub 2018 Jun 27. PMID: 29950720. |
| CYLD | Lork M, Verhelst K, Beyaert R. CYLD, A20 and OTULIN deubiquitinases in NF-κB signaling and cell death: so similar, yet so different. Cell Death Differ. 2017 Jul;24(7):1172-1183. doi: 10.1038/cdd.2017.46. Epub 2017 Mar 31. PMID: 28362430; PMCID: PMC5520167. |
| USP22 | Roedig J, Kowald L, Juretschke T, Karlowitz R, Ahangarian Abhari B, Roedig H, Fulda S, Beli P, van Wijk SJ. USP22 controls necroptosis by regulating receptor-interacting protein kinase 3 ubiquitination. EMBO Rep. 2021 Feb 3;22(2):e50163. doi: 10.15252/embr.202050163. Epub 2020 Dec 28. PMID: 33369872; PMCID: PMC7857539. |
| MAP3K7 | Goodall ML, Fitzwalter BE, Zahedi S, Wu M, Rodriguez D, Mulcahy-Levy JM, Green DR, Morgan M, Cramer SD, Thorburn A. The Autophagy Machinery Controls Cell Death Switching between Apoptosis and Necroptosis. Dev Cell. 2016 May 23;37(4):337-349. doi: 10.1016/j.devcel.2016.04.018. PMID: 27219062; PMCID: PMC4886731. |
| SQSTM1 | Goodall ML, Fitzwalter BE, Zahedi S, Wu M, Rodriguez D, Mulcahy-Levy JM, Green DR, Morgan M, Cramer SD, Thorburn A. The Autophagy Machinery Controls Cell Death Switching between Apoptosis and Necroptosis. Dev Cell. 2016 May 23;37(4):337-349. doi: 10.1016/j.devcel.2016.04.018. PMID: 27219062; PMCID: PMC4886731. |
| STAT3 | Wang X, Shao X, Gu L, Jiang K, Wang S, Chen J, Fang J, Guo X, Yuan M, Shi J, Ding C, Meng S, Xu Q. Targeting STAT3 enhances NDV-induced immunogenic cell death in prostate cancer cells. J Cell Mol Med. 2020 Apr;24(7):4286-4297. doi: 10.1111/jcmm.15089. Epub 2020 Feb 26. PMID: 32100392; PMCID: PMC7171322. |
| DIABLO | McComb S, Aguadé-Gorgorió J, Harder L, Marovca B, Cario G, Eckert C, Schrappe M, Stanulla M, von Stackelberg A, Bourquin JP, Bornhauser BC. Activation of concurrent apoptosis and necroptosis by SMAC mimetics for the treatment of refractory and relapsed ALL. Sci Transl Med. 2016 May 18;8(339):339ra70. doi: 10.1126/scitranslmed.aad2986. PMID: 27194728. |
| DNMT1 | Smith AD, Lu C, Payne D, Paschall AV, Klement JD, Redd PS, Ibrahim ML, Yang D, Han Q, Liu Z, Shi H, Hartney TJ, Nayak-Kapoor A, Liu K. Autocrine IL6-Mediated Activation of the STAT3-DNMT Axis Silences the TNFα-RIP1 Necroptosis Pathway to Sustain Survival and Accumulation of Myeloid-Derived Suppressor Cells. Cancer Res. 2020 Aug 1;80(15):3145-3156. doi: 10.1158/0008-5472.CAN-19-3670. Epub 2020 Jun 17. PMID: 32554751; PMCID: PMC7416440. |
| CFLAR | Fiore A, Ugel S, De Sanctis F, Sandri S, Fracasso G, Trovato R, Sartoris S, Solito S, Mandruzzato S, Vascotto F, Hippen KL, Mondanelli G, Grohmann U, Piro G, Carbone C, Melisi D, Lawlor RT, Scarpa A, Lamolinara A, Iezzi M, Fassan M, Bicciato S, Blazar BR, Sahin U, Murray PJ, Bronte V. Induction of immunosuppressive functions and NF-κB by FLIP in monocytes. Nat Commun. 2018 Dec 5;9(1):5193. doi: 10.1038/s41467-018-07654-4. PMID: 30518925; PMCID: PMC6281604. |
| BRAF | Najafov A, Zervantonakis IK, Mookhtiar AK, Greninger P, March RJ, Egan RK, Luu HS, Stover DG, Matulonis UA, Benes CH, Yuan J. BRAF and AXL oncogenes drive RIPK3 expression loss in cancer. PLoS Biol. 2018 Aug 29;16(8):e2005756. doi: 10.1371/journal.pbio.2005756. PMID: 30157175; PMCID: PMC6114281. |
| AXL | Najafov A, Zervantonakis IK, Mookhtiar AK, Greninger P, March RJ, Egan RK, Luu HS, Stover DG, Matulonis UA, Benes CH, Yuan J. BRAF and AXL oncogenes drive RIPK3 expression loss in cancer. PLoS Biol. 2018 Aug 29;16(8):e2005756. doi: 10.1371/journal.pbio.2005756. PMID: 30157175; PMCID: PMC6114281. |
| ID1 | Tan HY, Wang N, Chan YT, Zhang C, Guo W, Chen F, Zhong Z, Li S, Feng Y. ID1 overexpression increases gefitinib sensitivity in non-small cell lung cancer by activating RIP3/MLKL-dependent necroptosis. Cancer Lett. 2020 Apr 10;475:109-118. doi: 10.1016/j.canlet.2020.01.025. Epub 2020 Jan 28. PMID: 32004572. |
| CDKN2A | Medrano RFV, Hunger A, Catani JPP, Strauss BE. Uncovering the immunotherapeutic cycle initiated by p19Arf and interferon-β gene transfer to cancer cells: An inducer of immunogenic cell death. Oncoimmunology. 2017 May 19;6(7):e1329072. doi: 10.1080/2162402X.2017.1329072. PMID: 28811972; PMCID: PMC5543818. |
| HSPA4 | Johnston AN, Ma Y, Liu H, Liu S, Hanna-Addams S, Chen S, Chen C, Wang Z. Necroptosis-blocking compound NBC1 targets heat shock protein 70 to inhibit MLKL polymerization and necroptosis. Proc Natl Acad Sci U S A. 2020 Mar 24;117(12):6521-6530. doi: 10.1073/pnas.1916503117. Epub 2020 Mar 10. PMID: 32156734; PMCID: PMC7104336. |
| BCL2 | Lee KM, Lee H, Han D, Moon WK, Kim K, Oh HJ, Choi J, Hwang EH, Kang SE, Im SA, Lee KH, Ryu HS. Combined the SMAC mimetic and BCL2 inhibitor sensitizes neoadjuvant chemotherapy by targeting necrosome complexes in tyrosine aminoacyl-tRNA synthase-positive breast cancer. Breast Cancer Res. 2020 Nov 25;22(1):130. doi: 10.1186/s13058-020-01367-7. PMID: 33239070; PMCID: PMC7687715. |
| STUB1 | Tang MB, Li YS, Li SH, Cheng Y, Zhang S, Luo HY, Mao CY, Hu ZW, Schisler JC, Shi CH, Xu YM. Anisomycin prevents OGD-induced necroptosis by regulating the E3 ligase CHIP. Sci Rep. 2018 Apr 23;8(1):6379. doi: 10.1038/s41598-018-24414-y. PMID: 29686306; PMCID: PMC5913227. |
| FLT3 | Hillert LK, Bettermann-Bethge K, Nimmagadda SC, Fischer T, Naumann M, Lavrik IN. Targeting RIPK1 in AML cells carrying FLT3-ITD. Int J Cancer. 2019 Sep 15;145(6):1558-1569. doi: 10.1002/ijc.32246. Epub 2019 Mar 23. PMID: 30828789. |
| HAT1 | Carafa V, Nebbioso A, Cuomo F, Rotili D, Cobellis G, Bontempo P, Baldi A, Spugnini EP, Citro G, Chambery A, Russo R, Ruvo M, Ciana P, Maravigna L, Shaik J, Radaelli E, De Antonellis P, Tarantino D, Pirolli A, Ragno R, Zollo M, Stunnenberg HG, Mai A, Altucci L. RIP1-HAT1-SIRT Complex Identification and Targeting in Treatment and Prevention of Cancer. Clin Cancer Res. 2018 Jun 15;24(12):2886-2900. doi: 10.1158/1078-0432.CCR-17-3081. Epub 2018 Mar 13. PMID: 29535128. |
| SIRT2 | Carafa V, Nebbioso A, Cuomo F, Rotili D, Cobellis G, Bontempo P, Baldi A, Spugnini EP, Citro G, Chambery A, Russo R, Ruvo M, Ciana P, Maravigna L, Shaik J, Radaelli E, De Antonellis P, Tarantino D, Pirolli A, Ragno R, Zollo M, Stunnenberg HG, Mai A, Altucci L. RIP1-HAT1-SIRT Complex Identification and Targeting in Treatment and Prevention of Cancer. Clin Cancer Res. 2018 Jun 15;24(12):2886-2900. doi: 10.1158/1078-0432.CCR-17-3081. Epub 2018 Mar 13. PMID: 29535128. |
| SIRT1 | Carafa V, Nebbioso A, Cuomo F, Rotili D, Cobellis G, Bontempo P, Baldi A, Spugnini EP, Citro G, Chambery A, Russo R, Ruvo M, Ciana P, Maravigna L, Shaik J, Radaelli E, De Antonellis P, Tarantino D, Pirolli A, Ragno R, Zollo M, Stunnenberg HG, Mai A, Altucci L. RIP1-HAT1-SIRT Complex Identification and Targeting in Treatment and Prevention of Cancer. Clin Cancer Res. 2018 Jun 15;24(12):2886-2900. doi: 10.1158/1078-0432.CCR-17-3081. Epub 2018 Mar 13. PMID: 29535128. |
| PLK1 | Deeraksa A, Pan J, Sha Y, Liu XD, Eissa NT, Lin SH, Yu-Lee LY. Plk1 is upregulated in androgen-insensitive prostate cancer cells and its inhibition leads to necroptosis. Oncogene. 2013 Jun 13;32(24):2973-83. doi: 10.1038/onc.2012.309. Epub 2012 Aug 13. PMID: 22890325; PMCID: PMC3499666. |
| MPG | Allocca M, Corrigan JJ, Mazumder A, Fake KR, Samson LD. Inflammation, necrosis, and the kinase RIP3 are key mediators of AAG-dependent alkylation-induced retinal degeneration. Sci Signal. 2019 Feb 12;12(568):eaau9216. doi: 10.1126/scisignal.aau9216. PMID: 30755477; PMCID: PMC7150588. |
| BACH2 | Petanidis S, Domvri K, Porpodis K, Anestakis D, Freitag L, Hohenforst-Schmidt W, Tsavlis D, Zarogoulidis K. Inhibition of kras-derived exosomes downregulates immunosuppressive BACH2/GATA-3 expression via RIP-3 dependent necroptosis and miR-146/miR-210 modulation. Biomed Pharmacother. 2020 Feb;122:109461. doi: 10.1016/j.biopha.2019.109461. Epub 2019 Dec 30. PMID: 31918262. |
| GATA3 | Petanidis S, Domvri K, Porpodis K, Anestakis D, Freitag L, Hohenforst-Schmidt W, Tsavlis D, Zarogoulidis K. Inhibition of kras-derived exosomes downregulates immunosuppressive BACH2/GATA-3 expression via RIP-3 dependent necroptosis and miR-146/miR-210 modulation. Biomed Pharmacother. 2020 Feb;122:109461. doi: 10.1016/j.biopha.2019.109461. Epub 2019 Dec 30. PMID: 31918262. |
| MYCN | Nicolai S, Pieraccioli M, Peschiaroli A, Melino G, Raschellà G. Neuroblastoma: oncogenic mechanisms and therapeutic exploitation of necroptosis. Cell Death Dis. 2015 Dec 3;6(12):e2010. doi: 10.1038/cddis.2015.354. PMID: 26633716; PMCID: PMC4720889. |
| ALK | Nicolai S, Pieraccioli M, Peschiaroli A, Melino G, Raschellà G. Neuroblastoma: oncogenic mechanisms and therapeutic exploitation of necroptosis. Cell Death Dis. 2015 Dec 3;6(12):e2010. doi: 10.1038/cddis.2015.354. PMID: 26633716; PMCID: PMC4720889. |
| ATRX | Nicolai S, Pieraccioli M, Peschiaroli A, Melino G, Raschellà G. Neuroblastoma: oncogenic mechanisms and therapeutic exploitation of necroptosis. Cell Death Dis. 2015 Dec 3;6(12):e2010. doi: 10.1038/cddis.2015.354. PMID: 26633716; PMCID: PMC4720889. |
| TERT | Nicolai S, Pieraccioli M, Peschiaroli A, Melino G, Raschellà G. Neuroblastoma: oncogenic mechanisms and therapeutic exploitation of necroptosis. Cell Death Dis. 2015 Dec 3;6(12):e2010. doi: 10.1038/cddis.2015.354. PMID: 26633716; PMCID: PMC4720889. |
| SLC39A7 | Fauster A, Rebsamen M, Willmann KL, César-Razquin A, Girardi E, Bigenzahn JW, Schischlik F, Scorzoni S, Bruckner M, Konecka J, Hörmann K, Heinz LX, Boztug K, Superti-Furga G. Systematic genetic mapping of necroptosis identifies SLC39A7 as modulator of death receptor trafficking. Cell Death Differ. 2019 Jun;26(6):1138-1155. doi: 10.1038/s41418-018-0192-6. Epub 2018 Sep 20. PMID: 30237509; PMCID: PMC6748104. |
| SPATA2 | Kupka S, De Miguel D, Draber P, Martino L, Surinova S, Rittinger K, Walczak H. SPATA2-Mediated Binding of CYLD to HOIP Enables CYLD Recruitment to Signaling Complexes. Cell Rep. 2016 Aug 30;16(9):2271-80. doi: 10.1016/j.celrep.2016.07.086. Epub 2016 Aug 18. PMID: 27545878; PMCID: PMC5009064. |
| RNF31 | Kupka S, De Miguel D, Draber P, Martino L, Surinova S, Rittinger K, Walczak H. SPATA2-Mediated Binding of CYLD to HOIP Enables CYLD Recruitment to Signaling Complexes. Cell Rep. 2016 Aug 30;16(9):2271-80. doi: 10.1016/j.celrep.2016.07.086. Epub 2016 Aug 18. PMID: 27545878; PMCID: PMC5009064. |
| IDH1 | Yang Z, Jiang B, Wang Y, Ni H, Zhang J, Xia J, Shi M, Hung LM, Ruan J, Mak TW, Li Q, Han J. 2-HG Inhibits Necroptosis by Stimulating DNMT1-Dependent Hypermethylation of the RIP3 Promoter. Cell Rep. 2017 May 30;19(9):1846-1857. doi: 10.1016/j.celrep.2017.05.012. PMID: 28564603. |
| IDH2 | Yang Z, Jiang B, Wang Y, Ni H, Zhang J, Xia J, Shi M, Hung LM, Ruan J, Mak TW, Li Q, Han J. 2-HG Inhibits Necroptosis by Stimulating DNMT1-Dependent Hypermethylation of the RIP3 Promoter. Cell Rep. 2017 May 30;19(9):1846-1857. doi: 10.1016/j.celrep.2017.05.012. PMID: 28564603. |
| KLF9 | Tung B, Ma D, Wang S, Oyinlade O, Laterra J, Ying M, Lv SQ, Wei S, Xia S. Krüppel-like factor 9 and histone deacetylase inhibitors synergistically induce cell death in glioblastoma stem-like cells. BMC Cancer. 2018 Oct 22;18(1):1025. doi: 10.1186/s12885-018-4874-8. PMID: 30348136; PMCID: PMC6198521. |
| HDAC9 | Tung B, Ma D, Wang S, Oyinlade O, Laterra J, Ying M, Lv SQ, Wei S, Xia S. Krüppel-like factor 9 and histone deacetylase inhibitors synergistically induce cell death in glioblastoma stem-like cells. BMC Cancer. 2018 Oct 22;18(1):1025. doi: 10.1186/s12885-018-4874-8. PMID: 30348136; PMCID: PMC6198521. |
| HSP90AA1 | Yan C, Oh JS, Yoo SH, Lee JS, Yoon YG, Oh YJ, Jang MS, Lee SY, Yang J, Lee SH, Kim HY, Yoo YH. The targeted inhibition of mitochondrial Hsp90 overcomes the apoptosis resistance conferred by Bcl-2 in Hep3B cells via necroptosis. Toxicol Appl Pharmacol. 2013 Jan 1;266(1):9-18. doi: 10.1016/j.taap.2012.11.001. Epub 2012 Nov 9. PMID: 23147571. |
| LEF1 | Liu P, Xu B, Shen W, Zhu H, Wu W, Fu Y, Chen H, Dong H, Zhu Y, Miao K, Xu W, Li J. Dysregulation of TNFα-induced necroptotic signaling in chronic lymphocytic leukemia: suppression of CYLD gene by LEF1. Leukemia. 2012 Jun;26(6):1293-300. doi: 10.1038/leu.2011.357. Epub 2011 Dec 13. PMID: 22157808. |
| BNIP3 | Kim JY, Kim YJ, Lee S, Park JH. BNip3 is a mediator of TNF-induced necrotic cell death. Apoptosis. 2011 Feb;16(2):114-26. doi: 10.1007/s10495-010-0550-4. PMID: 20963496. |
| CD40 | Qiu X, Klausen C, Cheng JC, Leung PC. CD40 ligand induces RIP1-dependent, necroptosis-like cell death in low-grade serous but not serous borderline ovarian tumor cells. Cell Death Dis. 2015 Aug 27;6(8):e1864. doi: 10.1038/cddis.2015.229. PMID: 26313915; PMCID: PMC4558516. |
| BCL2L11 | Locatelli SL, Cleris L, Stirparo GG, Tartari S, Saba E, Pierdominici M, Malorni W, Carbone A, Anichini A, Carlo-Stella C. BIM upregulation and ROS-dependent necroptosis mediate the antitumor effects of the HDACi Givinostat and Sorafenib in Hodgkin lymphoma cell line xenografts. Leukemia. 2014 Sep;28(9):1861-71. doi: 10.1038/leu.2014.81. Epub 2014 Feb 24. PMID: 24561519. |
| EGFR | Zhao Q, Kretschmer N, Bauer R, Efferth T. Shikonin and its derivatives inhibit the epidermal growth factor receptor signaling and synergistically kill glioblastoma cells in combination with erlotinib. Int J Cancer. 2015 Sep 15;137(6):1446-56. doi: 10.1002/ijc.29483. Epub 2015 Mar 6. PMID: 25688715. |
| DDX58 | Dunker W, Ye X, Zhao Y, Liu L, Richardson A, Karijolich J. TDP-43 prevents endogenous RNAs from triggering a lethal RIG-I-dependent interferon response. Cell Rep. 2021 Apr 13;35(2):108976. doi: 10.1016/j.celrep.2021.108976. PMID: 33852834; PMCID: PMC8109599. |
| TARDBP | Dunker W, Ye X, Zhao Y, Liu L, Richardson A, Karijolich J. TDP-43 prevents endogenous RNAs from triggering a lethal RIG-I-dependent interferon response. Cell Rep. 2021 Apr 13;35(2):108976. doi: 10.1016/j.celrep.2021.108976. PMID: 33852834; PMCID: PMC8109599. |
| APP | Wang L, Shen Q, Liao H, Fu H, Wang Q, Yu J, Zhang W, Chen C, Dong Y, Yang X, Guo Q, Zhang J, Zhang J, Zhang W, Lin H, Duan Y. Multi-Arm PEG/Peptidomimetic Conjugate Inhibitors of DR6/APP Interaction Block Hematogenous Tumor Cell Extravasation. Adv Sci (Weinh). 2021 Jun;8(11):e2003558. doi: 10.1002/advs.202003558. Epub 2021 Mar 18. PMID: 34105277; PMCID: PMC8188212. |
| TNFRSF21 | Wang L, Shen Q, Liao H, Fu H, Wang Q, Yu J, Zhang W, Chen C, Dong Y, Yang X, Guo Q, Zhang J, Zhang J, Zhang W, Lin H, Duan Y. Multi-Arm PEG/Peptidomimetic Conjugate Inhibitors of DR6/APP Interaction Block Hematogenous Tumor Cell Extravasation. Adv Sci (Weinh). 2021 Jun;8(11):e2003558. doi: 10.1002/advs.202003558. Epub 2021 Mar 18. PMID: 34105277; PMCID: PMC8188212. |
